# Characterization of dengue virus in *Aedes aegypti* and *Aedes albopictus *spp. of mosquitoes: A study in Khyber Pakhtunkhwa, Pakistan

**DOI:** 10.22099/mbrc.2018.29073.1315

**Published:** 2018-06

**Authors:** Hussain Mubbashir, Shahzad Munir, Rahim Kashif, Haider Bashir Nawaz, Basit Abdul, Khattak Baharullah

**Affiliations:** 1 Vector Borne Diseases Lab, Department of Microbiology, Kohat University of Science and Technology, Kohat, Khyber Pakhtunkhwa, 26000 Pakistan; 2 Faculty of Plant Protection, Yunnan Agricultural University, Kunming 650201, Yunnan, China; 3 Beijing Key Laboratory of Genetic Engineering Drug and Biotechnology, Institute of Biochemistry and Biotechnology, College of Life Sciences, Beijing Normal University, Beijing 100875, China

**Keywords:** Dengue Virus, Aedes mosquitoes, Khyber Pakhtunkhwa Pakistan

## Abstract

Dengue is a vector-borne disease caused by dengue virus. According to the recent report of CDC that one-third population of the world are at high risk with Dengue fever. The prevalence of the dengue hemorrhagic fever was found more in tropical and sub-tropical regions of the world. *Aedes* mosquitoes was reported as the main cause of transmission of dengue virus. So the current study was planned to characterize the virus in *Aedes* mosquitoes collected from different area of Pakistan. In current investigation, *Aedes* mosquitoes and larvae were trapped under conducive conditions which are counted as 495 *Aedes* mosquitoes and 260 *Aedes* larvae. First of all, adult mosquitoes were identified morphologically under microscopy, counted as 73.3% *Ae. aegypti* and 26.7% *Ae. albopictus*. Finally, reverse transcriptase polymerase chain reaction analyses that only 4 adults of *Aedes* mosquitoes and 10 *Aedes* larvae as naturally infected with dengue virus with possible source *Ae. aegypti*. This study basically uncovered the presence of virus in different species of mosquitoes in southern regions of Pakistan. The present study will also give us an insight for vector control programs of dengue virus in the affected area.

## INTRODUCTION

With more than one-third of the world’s population living in areas at risk for infection, dengue virus is a leading cause of illness and death in the tropics and subtropics. As many as 400 million people are infected yearly. Dengue fever is Vector born disease and caused by any one of four related viruses transmitted by mosquitoes (CDC). Mosquitoes are medically and economically significant group of insects among dipterans that serves as vector for a various number of human and zoonotic disease pathogens [1]. *Aedes aegypti* is a competent vector of mosquito-borne diseases and is the primary transmitter of yellow fever, Zika, Chikungunya, and dengue viruses [2], causing 50 million cases of infection and 300,000 deaths each year in tropical and subtropical areas [1, 3]. The Asian tiger mosquito, *Aedes albopictus* is currently an important vector for dengue and its role in the transmission of arthropod-borne viruses’ arboviruses. Although, its vectorial capacity for dengue virus (DENV) is lower than that of *Ae. Aegypti* while *Ae. albopictus* has been responsible for dengue epidemics in Japan, Malaysia, China and Hawaii [4]. In the late autumn of 2014, *Ae. albopictus* was ﬁrst reported in the Sistan and Baluchestan Province of Iran in the southeast of the country bordering Pakistan [5]. Infection can be asymptomatic but can also lead to life-threatening dengue hemorrhagic fever (DHF) or dengue shock syndrome [6]. The first serologically confirmed outbreak of DF in Pakistan was reported in 1994 [7], during the last 5 years, situation of dengue viral transmission has been worsening in Pakistan, especially in the post-monsoon period, with densely populated cities like Karachi and Lahore being under severe threat of dengue epidemics [8]. During 2012-2016, 41,311 cases were reported in Pakistan; out of these, almost 37% of the cases were reported from Khyber Pakhtunkhwa, 28% from Punjab, 27% from Sindh, and 8% from Baluchistan [9]. As there is no effective medicine and vaccine available, vector control remains the most effective measure to prevent its transmission and outbreak [10]. DENV-1 and 2, were identified during 1994 outbreak while DENV-3 was reported firstly in 2005–2006 outbreak, from 2007-2009, DENV-2 and 3 were reported during the outbreaks in Lahore while showing revitalization in 2010 (Ali et al. 2013), especially in Punjab, Sindh and some cities in the Khyber Pakhtunkhwa regions after floods occurring; most prevalent serotypes were DENV-2 and 3 but also with a lower occurrence of DENV-4 [11]. KPK province is located in the north-western region of the country with an area of 74,521 km^2^ and a population of 26.9 million; in 2013, KPK was hit by a major dengue outbreak infecting more than 11,000 individuals, mainly in the Swat district and neighboring areas, including the Malakand, Kohat, and Mansehra districts [12]. The objective of this study was the detection of dengue virus and its serotype in naturally infected *Aedes* mosquitoes from different areas southern Khyber-Pakhtunkhwa by reverse transcriptase polymerase chain reaction (RT-PCR).

## MATERIALS AND METHODS

The present study was conducted in different areas of district Kohat latitude of 33,5869 (3335'12.840"N) and at a longitude of 71,4414 (7126'29.040"E) with an altitude of 489 meters (1,604 feet), Khyber Pakhtunkhwa from the month of June 2015 to June 2016. In district Kohat, during the month of August in summer and December in winter, maximum humidity (67%) has been reported. *Aedes* mosquitoes were trapped from different rural and urban areas of the study district before sunset and after sunrise, using Centers for Disease Control and Prevention (CDC) light traps. CDC traps were hanged at the height of about 6 feet from earth surface in places near plants, trees or containers having stagnant water. 

The collected mosquitoes were placed in 70% ethanol. Different pots (containers, jars, bottles etc.) were also kept under observation for *Aedes* mosquito eggs, larvae, pupae, and adults. Collected adults and larvae of *Aedes* mosquitoes were placed in 70% ethanol brought to Vector-borne Disease Management center at Kohat University of Science and Technology (KUST), Khyber Pakhtunkhwa, Pakistan. Adults of *Ae. Aegypti* and *Ae. albopictus* were separated from other mosquitoes by their morphological characteristics under microscope.

RNA from adults and larvae of *Aedes* mosquitoes were extracted by Favor PrepTM Viral Nucleic Acid Extraction Kit I according to manufacturer instructions. RNA was stored at -20 °C for further use. The same protocol was repeated for *Aedes* larvae. RNA extracted from trapped *Aedes* mosquitoes and larvae, was converted to cDNA by using reverse transcriptase enzyme. cDNA was then pooled (cDNA of 20 mosquitoes per pool) separately for adults and larvae, reactions were performed. Reverse transcriptase enzyme and dengue virus reverse primer D2 were used to convert target sequence of the virus RNA into the complimentary copy of DNA (cDNA). After that forward dengue virus primer, D1 was used for amplification of cDNA. In 13 μl of RT mixture, 12 μl of extracted RNA was added. 5×RT buffer was consisted of Tris-HCl =260 mM, pH = 8.4, KCl = 370 mM, MgCl2 = 20 mM, dNTP’s = 1.3mM of each, D2 primer = 30 pmol, Superscript II= 250 units, RNase inhibitor = 20 units. This mixture was incubated at 37°C for 60 minutes and cDNA was synthesized. The enzyme was inactivated at 100°C for 10 minutes. Further, 50 μl volume was used for the first round of PCR. In 38 μl of PCR mixture, 12 μl of cDNA were added.

PCR was then performed in a 50 mL reaction volume using a set of five primers comprising a dengue virus consensus reverse primer and four serotype specific forward primers. The reaction contained of initial denaturation at 94°C for 5 minutes followed by 35 cycles (denaturation at 94°C for 1 minute, annealing at 55°C for 90 sec and extension at 72°C for 2.5 minutes) and final extension for 7 minutes at 72°C and at last hold at 4°C. Regions of four serotypes that are, 482 bp of DENV-1, 119 bp of DENV-2, 290 bp of DENV-3 and 392 bp of DENV-4 were amplified by D1 primer. With the help of 1.8% agarose gel electrophoresis, amplified fragments of cDNA of dengue virus were detected.

## RESULTS AND DISCUSSION

A total of 1150 mosquitoes were trapped from different locations of district Kohat by using CDC traps. Among them, 495 were *Aedes* mosquitoes. The highest number of mosquitoes was trapped during the month of August (data not shown); heavy rainfall occurs that causes the mosquitoes to grow in stagnant water. *Aedes *mosquitoes were trapped from different containers where water can be naturally stored including pots (jars, bottles, pitchers etc), unused buckets, drums, tyres, tree holes and cemented water reservoirs. These containers were already placed near patient's houses, poultry farms, dairy farms, and in open-aired areas. Previous studies reported the same results [13-17].

For the molecular study, RT-PCR of these pools was performed for amplification of dengue virus genome. RT-PCR results were positive for only four pools of adult *Aedes* mosquito samples (Table 1). And also for *Aedes *larvae samples, four pools show positive results and remaining pool's results were negative (Table 2). 

**Table1:** RT-PCR results for adult mosquito's pools

**S. No**	**Sample No**	**Pool No (20 mosquitoes per pool)**	**RT-PCR results**
1	1-20	1	+
2	21-40	2	-
3	41-60	3	+
4	61-80	4	+
5	81-100	5	-
6	101-120	6	-
7	121-140	7	-
8	141-160	8	-
9	161-180	9	-
10	181-200	10	-
11	201-220	11	-
12	221-240	12	+
13	241-260	13	-
14	261-280	14	-
15	281-300	15	-

**Figure 1:**
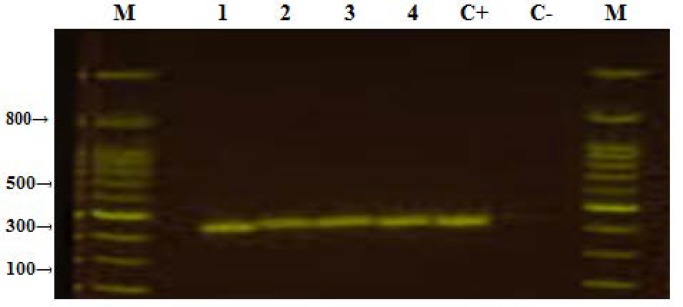
DEN-3 serotype of *Aedes* mosquitoes with 290 bp; From 1 to 4 all are positive, M is the Marker, Lane 5 (C+) is the positive control and Lane 6 (C-) is the negative control

As RT-PCR result was positive for the pool no 1, 3, 4 and 12 of adult mosquitoes and the pool no 1, 2 5 and 6 of *Aedes* mosquito larvae so RT-PCR was again performed for each sample of these positive pools. RT-PCR then showed positive results for adult's sample no 16 of pool no 1, sample no 44 of pool 3 and sample no 63 of pool 4 that were collected from Lachi and sample no 235 of pool no 12 that was collected from Bahaderkot. For larvae RT-PCR results were positive for sample no 4, 9, 11 and 16 of pool no 1 collected from Lachi, sample no 21, 25 and 33 of pool no 2 collected from Lachi, sample no 92 of pool no 5 collected from College Town and sample no 101 and 107 of pool no 6 collected from Bahaderkot. Dengue virus with RT-PCR positive results are displayed in the Figure 1. 

**Table 2:** RT-PCR results for mosquito's larvae pools

**S. No**	**Sample No**	**Pool No **	**RT-PCR results**
1	1-20	1	+
2	21-40	2	+
3	41-60	3	-
4	61-80	4	-
5	81-100	5	+
6	101-120	6	+
7	121-140	7	-
8	141-160	8	-
9	161-180	9	-
10	181-200	10	-
11	201-220	11	-
12	221-240	12	-
13	241-260	13	-

Tyres act as a source of transport of dengue vectors (*Aedes *mosquitoes) not only within the country but also in different parts of the world because *Aedes* mosquitoes may breed in tyres if store in the water [18] and according to our study, tyres may be a factor of the outbreak in Lachi. In district, Kohat people mostly store water in different containers so *Aedes* mosquitoes easily breed there. That‘s why different areas of district Kohat, mosquitoes are increasing in number and can also disperse to other areas also is in close agreement with [19]. We point out that collected *Aedes* mosquitoes were identified as 61% female *Aedes* mosquitoes and 39% male *Aedes* mosquitoes of both species (*Ae. aegypti* and *Ae. albopictus*) which indicated that female *Aedes* mosquitoes were in higher number than male mosquitoes. This may be due to their blood feeding behavior from humans to complete life cycle. Similar observations have been observed by [20].
